# The politics of staying behind the frontline of coronavirus

**DOI:** 10.12688/wellcomeopenres.15964.2

**Published:** 2020-09-02

**Authors:** Stephanie Davies

**Affiliations:** 1 Department of Psychosocial Studies, Birkbeck University of London, London, WC1B 5DT, UK

**Keywords:** COVID-19 pandemic, NHS culture, ethnography of care, Dejours, temporality and healthcare

## Abstract

Intended as a contribution to the *Waiting in Pandemic Times* project Collection in response to COVID-19, this short theoretical paper views the coronavirus crisis in terms of its unpredictable effects on the interior life of the National Health Service (NHS) workforce. Written immediately following the suspension (due to the pandemic) of an ethnographic investigation of waiting in a general practice in London, it tracks the first signs that working definitions of time would struggle to survive the onset of a temporality of acute crisis in the NHS. The paper considers what it might mean for healthcare practitioners at this particular moment in the NHS’s history to be living through an experience of ‘the ordinary’ breaking down. It also follows hints of new temporal modes of care appearing during this same period when one kind of crisis in the NHS has been put on hold, and another (the crisis of coronavirus) is just getting underway.

During a crisis on the scale of coronavirus, the invisible forces holding an institution’s ‘time’ in place ( Hammer, 2011) ^1^ can seem to collapse, as they suddenly lose their power to regulate the institution’s everyday life in the drastically altered situation. I was a researcher undertaking an investigation of waiting in a London general practice when I heard the news that the virus, seemingly so far away, had arrived, having entered literally through the front door. In the hours that followed the first suspected case, all thoughts were immediately drawn towards the virus, siphoned off from wherever they had happened to be only an hour before and, after having being relied upon for so long to replenish everyday life in the surgery with its essential quality of ‘everydayness’, many long established orderings of time and space – meeting times, consultation times, waiting room times, lunch-time, opening and closing times, patterns of movement through the building - were reduced to just the remainders of what was left over after the coronavirus had taken hold. So too were the ordinary patterns of behaviour and identification that had belonged to them; of employees knowing how to compose themselves as those who ‘work for the National Health Service (NHS)’. Through rapid modes of improvisation and with an intensity that might have come from the combined energies of so many people all furiously channelling what was going on around them, they very quickly began to reconstitute themselves as those who work for a different kind of NHS, one still in the process of being formed out of a time of national emergency. This all seemed to take place over the course of a single morning, just a day after the first suspected case of coronavirus had entered the building.

The original research questions I had just then begun to investigate had sought to grasp *waiting in healthcare* in its everyday aspect — as a circumstance so taken for granted that many of its lesser known functions for general practice tended to be overlooked. Through observations of patient consultations and other sites of decision-making, I had been watching for the details of how waiting happens in these settings, whether out of necessity, by accident or by design. I wanted to know what people ‘did’ with waiting: how they used or consumed it; how waiting was ‘made’ or engineered out of the things available and at hand; what it ‘looked like’ to wait here, and how waiting was being made ‘readable’ or ‘unreadable’ for the NHS or for this particular organisation. When the virus had struck without warning just as the observations had started, the structures that had supported everyday forms of waiting appeared to fall away, carrying my original research questions along with them. In place of my old investigation of waiting at its most ordinary in general practice, the new questions arising had corresponded to an altered reality — one not at all orderable by the standards of the everyday. 

The orderings of time that are usually seen to be of utmost importance for the future – recording, inspecting, reviewing for instance – are suddenly made to appear extremely relative alongside the infinitely more pressing and immediate demands of the present: the saving of life, the protecting of one’s own life, and the need to survive the crisis. At such a moment, a person might get caught up in the end of one set of working conceptions of time, before another has even begun to circulate. Yet, for those healthcare workers who might now be experiencing something like this in their own NHS workplaces, the failure of the ordinary to assimilate the fears, risks and demands connected with an unknown virus, is an event that they have had no choice but to find some way of working through. So how are they keeping time in such a crisis? ( Catty, 2020, *Waiting in Pandemic Times*).

The interior life of the workplace is said by Christophe Dejours to hardly ever be allowed to show itself, except in very rare situations ( Dejours, 2007). The hiddenness of its invisible inner workings and affective economies is believed to be partly due to a lack of interest by the public whose currency is mainly that of the name or brand of the organisation (the part that faces outwards), but also because the workers themselves are thought to be complicit in secreting the inner-functioning of their own institution with whom they are required, at least in part, to identify. At this time of great public interest in watching the collision of coronavirus with healthcare systems all over the world, the growing intensity of collective identification with the aims of the NHS and its projection onto the workforce, could mean that the privately felt realities for NHS staff, of becoming part of a temporality of crisis, are even more likely than usual to remain hidden from view.

On the surface, the crisis appears to have prompted new modes of agency that have begun to emerge in NHS settings hinting at a shift in the perspectives of its workforce. This is discernible in the celebration of activities and practices, that may not always have been considered reasonable for NHS employees to have engaged in before now. For example, carework that takes the form of a commitment to ‘staying with, in spite of’, has come to the fore in images and testimonials depicting doctors and nurses remaining steadfast at their posts, ‘heedless of their own health as they work tirelessly to care for people in the face of the Coronavirus pandemic’ ^2^. It is not clear whether such practices - of *staying* to care for others, correspond to the experiences of frontline NHS employees as a multiplicity, or whether they are imposed from elsewhere, or a combination of both, but because NHS staff are *seen* to be working on behalf of others at their own risk, and often in a way that requires them to withstand the most concentrated and contagious parts of the pandemic over an indefinite length of time, those who *stay* in post, or return to posts they had previously left, appear to be choosing to exercise a form of altruistic endurance. Moore (2020) notices that in social media posted by NHS staff bearing the message, ‘we stay here for you – please stay home for us’, the NHS assumes the form of ‘a subject that waits for citizens in their time of need’. Coming in the midst of a crisis, the outward forms for this kind of ‘extreme’ carework — of duty, self-sacrifice and a commitment to ‘staying with, in spite of’ might be read as the signs of a new orientation to the present characterised by the ‘making of promises’.

In Paul Ricoeur’s hermeneutic philosophy of time, the promise describes the public act of putting myself under an obligation of *doing* something, *for* someone. It is ‘the projection of a horizon of expectation’ made possible through the inscription of dialogue onto the public space where historical responsibility for it can be assumed’ ( Ricoeur, 1990, p. 234). Nomatter how unbelievable or fleeting the experiences of living and working through the first days of the pandemic might turn out to be in the longer term, the making of a promise stakes a claim in the present, announcing and prolonging it. There is something not quite congruent however, about the promise implied by such messages as ‘we stay here for you – please stay home for us’. When glimpsed from below the level of image and identification, practices of staying and of ‘being here for you’ need to be understood as coming after a much longer, drawn out time in which the normal working day has been organised around the assumption that care is something that can only be apprehended at the specular level of the organisation, and where time is reduced to being just one of the costs of its production. They hint at modes of care that have been inspired by affective investments in what is ‘real’ about care work during a pandemic - its resistance to appropriation as, just ‘a task to be accomplished’ ( Dejours & Deranty, 2010, p. 451) ^3^. Considering that healthcare of all kinds has, until now, tended to be seen as synchronic output; as happening all at once, with little attention to how a particular labour of care might evolve over time, or how a continued engagement with it might help to ‘sustain interdependent worlds’ ( Bellacasa, 2012, p. 198), the appearance of an unconditional promise to ‘stay here for you’ is something of an anomaly, pushing in the opposite direction to the one that healthcare had been going in.

Unpredictable, prolonged and intermittent timeframes are to be expected in the NHS, particularly in relation to chronic, multiple or undiagnosable illness of the kind that now makes up most of its workload. It could be the continuation of an older formulation of care as synchronic, that has made staying with the idea that the activity of care might still be worthwhile in and of itself, increasingly difficult to justify ( Latimer, 2000). Though outwardly they may be focused on making more ‘time for care’ ( NHS England, 2019), policy initiatives that have sought to curtail the patient’s and the practitioner’s experience of time passing have tended to result in many temporal practices of care being rendered as obsolete. This is not to say that discretional practices of offering more time to some patients are not one of the inevitable consequences of making the offer to care in the first place, or that they haven’t always gone on and won’t continue to do so. But nevertheless, the growing concern amongst clinicians over finding themselves unable to spend time on the things that matter most to themselves and their patients, has been met until now with a response that questions their ability ever to have really known what it was worth their while having cared about in the first place. As one NHS England consultant put it, ‘we wondered how health and care systems could design services that would improve peoples’ lives, if the people running the system didn’t understand what matters most?’.

Kathleen Stewart observes that ‘there’s a politics to being/feeling connected (or not), to affective contagion, and to all the forms of attunement and attachment’ ( Stewart, 2007, p. 16). It changes the way we think about the politics of staying at the frontline of a pandemic, if we remember that many of the most experienced members of the NHS workforce, those who have stayed or returned, and who are still working on ‘our’ behalf, bring with them a decades long history of attachment to the institution in relation to the slow collapse of a former symbolic order ^4^. This is an order they worked hard to delay the future collapse of, even though they might well have wished to be released from it. It is a situation that has proven to be unendurable for many, as the annual problem of how to fill all the vacancies in British general practice affirms. Each year, a steady stream of doctors under the age of fifty, have made the decision not to stay, not to carry on. More than just a feeling of exhaustion from overwork, by the time they have left, many of these people, described quite tragically in one study as the ‘lost to the NHS’ ( Doran *et al.*, 2016) have reached the point of feeling that they had in some way been locked out from the time when *real* care was taking place.

Superficially, I think it would be difficult to imagine a more complete reversal of the recent past than what we are seeing on this new frontline, where virtually everything that ‘the NHS’ *does* is seen as extremely valuable, heroic even, in its relation to a future that has yet to unfold. One of the most contagious forms of agency that a temporality of acute crisis seems to be able to offer us now, is a capacity to become so immersed in the *doings* of the present that nothing else seems to matter ^5^. In this respect, coronavirus appears to have achieved in hours, what the official long form strategies for ‘releasing time’ never could, which is to have forced the making of more time for care into the present. In fact, an orientation to *this time - as care*, has not so much been offered to the workforce as *required* of them, just as to a lesser extent, it has been required of everybody whose time is having to be lived out firmly in the present (in lockdown), so that others might have more time to live on.

As with the politics of any ‘surge’, the afterlife of this crisis and of all the time for care that has miraculously bubbled up around it, may depend on events that have yet to come to pass; where the surge ‘might go, what happens, how it plays itself out and in whose hands’ ( Stewart, 2007, p. 15). As my own observations of general practice were cut short, I was one of those who couldn’t stay to follow the energy, to see ‘where it might go’, but from what I did see of the frontline after the first shock, I would say that there is something very fragile and forgetful about the forms the ordinary is taking and what they have the power to release under the influence of crisis.

## Data availability

Any insights are based only partly and tangentially on ethnographic observations of a clinical site. No results are included in the article which might be said to be based on the raw data collected during these observations.
