# Supplementary material for: The politics of staying behind the frontline of coronavirus
Source: Wellcome Open Res. 2020 Sep 2;5:131. Originally published 2020 Jun 10. [Version 2] doi: 10.12688/wellcomeopenres.15964.2 (PMC7471624; doi:10.12688/wellcomeopenres.15964.2)
Supplement: Peer review history

# Peer review: Reviewer response for version 2

**DOI:** 10.21956/wellcomeopenres.17867.r40249

**Published:** 2020-09-03

**Authors:** Sara M. Bergstresser

**Affiliations:** 1 Columbia University, New York, NY, USA

You have successfully clarified the nature of your contribution. The detailed background of how the topic emerged by necessity is a valuable addition. Good work.

Is the work clearly and accurately presented and does it cite the current literature?

Partly

If applicable, is the statistical analysis and its interpretation appropriate?

Not applicable

Are all the source data underlying the results available to ensure full reproducibility?

No source data required

Is the study design appropriate and is the work technically sound?

Partly

Are the conclusions drawn adequately supported by the results?

Yes

Are sufficient details of methods and analysis provided to allow replication by others?

Partly

Reviewer Expertise:

Anthropology (cultural medical focus), Public Health, Epidemiology, Bioethics.

I confirm that I have read this submission and believe that I have an appropriate level of expertise to confirm that it is of an acceptable scientific standard.

# Peer review: Reviewer response for version 1

**DOI:** 10.21956/wellcomeopenres.17508.r39377

**Published:** 2020-07-21

**Authors:** Sara M. Bergstresser

**Affiliations:** 1 Columbia University, New York, NY, USA

This article topic is particularly timely and important for the current moment. The breakdown of ordinary, everyday routines is an under-examined aspect of how crises manifest in social and professional worlds. I think there is a great deal of promise here, but there is also room to improve the paper.

 This article offers an interesting glimpse into the theoretical analysis that would be used to contextualize an ethnographic study. My main concern (as someone trained in ethnographic research) is that for an article defined as being based in part on ethnographic observations, there is very little detailed ethnographic description included; the note indicates that “No results are included in the article which might be said to be based on the raw data collected during these observations”. It might be the case that there are practical limitations on what data can be divulged at this point in the study, but if that is the case, I would recommend reframing the article more clearly as a theoretical piece, which may still include the author’s personal narrative as auto-ethnographic portions.

 There are two distinct paths to improvement. First, it can better fulfill the promise of ethnographic research by including more ethnographic description and data. For example, it could include additional passages like the quote from the NHS England consultant on p. 4. Second, if the first option is not feasible, it could be reframed to clearly offer a primarily theoretical rather than an ethnographic reading of the topic. The instances of personal narrative and observations can be integrated in either case, and additional connection of the author’s personal experiences to the theoretical discussion would be productive.

 I recognize that ethnographic work often does not fit the assumptions of a quantitative statistical science framework, which is often seen as the default, and that this may be the limiting factor – i.e. that including data could expose the piece to field-inappropriate questions of reproducibility. I would hope that if this is the limiting factor, that those in charge of editorial oversight might find a way for ethnographic data to be discussed in a way that does not subject it to questions of quantitative reproducibility, as qualitative ethnography is an inherently different form of knowledge production.

Is the work clearly and accurately presented and does it cite the current literature?

Partly

If applicable, is the statistical analysis and its interpretation appropriate?

Not applicable

Are all the source data underlying the results available to ensure full reproducibility?

No source data required

Is the study design appropriate and is the work technically sound?

Partly

Are the conclusions drawn adequately supported by the results?

Yes

Are sufficient details of methods and analysis provided to allow replication by others?

Partly

Reviewer Expertise:

Anthropology (cultural medical focus), Public Health, Epidemiology, Bioethics.

I confirm that I have read this submission and believe that I have an appropriate level of expertise to confirm that it is of an acceptable scientific standard, however I have significant reservations, as outlined above.

# Response

**Published:** 2020-08-20

**Authors:** Stephanie Davies

**Affiliations:** Birkbeck College, UK

Thank you very much for your review of my article.  Your comments were gratefully received and read by me.  

I do understand your reservations over my inclusion of the term ‘ethnographic’ in the paper which as you correctly point out, is primarily theoretical in its orientation.  In response to your suggestion for either clarifying the theoretical nature of the work, or else adding in more material to support its claim to be ethnographic, I have included some statements which I hope will emphasise that this is intended as a theoretical rather than as an ethnographic article.  They include a description of the work in the abstract as a ‘short theoretical paper’ where I specify that it was written immediately following the suspension of my original ethnographic investigation of waiting in general practice. This is in addition to the note at the end stating that any insights referred to in the work are based only partly and tangentially on ethnographic observations. I’ve also added a paragraph providing an outline of my original ethnographic study which I hope will help to draw a distinction in the reader’s mind between the different pieces of work.

# Peer review: Reviewer response for version 1

**DOI:** 10.21956/wellcomeopenres.17508.r39039

**Published:** 2020-06-11

**Authors:** Trish Greenhalgh

**Affiliations:** 1 Department of Primary Care Health Sciences, University of Oxford, Oxford, UK

I didn't know about the 'Waiting in Pandemic Times' project until I was invited to review this paper. So it was a bit of a treat to find a reflexive, theory-rich essay (I'm a dual qualified researcher in both medicine and social sciences, and have been peer-reviewing about one paper per day since March, all of them fast-tracked clinical papers on diagnostic tests, treatments etc). 

 So I liked the idea.

 I also liked the essay, broadly. It conveys very well the impact COVID-19 had on general practice - the abrupt disruption of structured normality, with a particular focus on temporal structuring. Love the Ricoeur.

 How to make it better? 

 Firstly, it lacks a sense of audience. Who are you writing for? As an academic clinician whose time has been disrupted since early March (no days off, no evenings off, just work work work to try to fight the fire), I didn't feel like you were speaking TO me, more that you were speaking ABOUT me. I felt I didn't have time to slow down and do a close read of your paper - partly because it was an unnecessarily hard read (one sentence at least went on for 17 lines!). This could be easily addressed by writing in shorter, clearer sentences (see Annemarie Mol as an example of someone who conveys complex philosophical concepts in prose that is delightfully easy to follow). More than ever, sociologists and humanities people who seek to enlighten and enrich the worlds of clinicians need to put work into making their writing accessible. (and I write as someone with a foot in both camps!)

 Related to that point, and on the assumption that you do want to 'speak to' a healthcare audience, I strongly suggest you introduce some of your philosophical friends to that audience. Tell us just a bit about what Ricoeur said about time. It is clear to you (and I myself dimly remember) that Ricoeur placed great emphasis on the fact that we live in time and in history. Your observations about the disruption of time as the first COVID case appeared in GP land would be so much more powerful if you had brought the reader into Ricoeur's world with a brief intro. Likewise for the other greats you briefly refer to. 

 At the beginning I'd like to know more about this waiting project. Before COVID messed it up, what aspect of waiting were you studying? A paragraph with some ethnographic reflections on that study would convey what was canonical about general practice, making your subsequent description of disruption more powerful. 

 I wonder, too, whether you might return to that description of the canonical at the end of your essay and consider how far that has been lost forever (or for the foreseeable future)?

 Hope these comments help, and thanks for taking me into a fascinating set of reflections.

Is the work clearly and accurately presented and does it cite the current literature?

Partly

If applicable, is the statistical analysis and its interpretation appropriate?

Not applicable

Are all the source data underlying the results available to ensure full reproducibility?

No source data required

Is the study design appropriate and is the work technically sound?

Partly

Are the conclusions drawn adequately supported by the results?

Yes

Are sufficient details of methods and analysis provided to allow replication by others?

Partly

Reviewer Expertise:

GP, social scientist.

I confirm that I have read this submission and believe that I have an appropriate level of expertise to confirm that it is of an acceptable scientific standard.
